# Age Is an Effect Modifier of Thrombectomy Benefit in a Socioeconomically Vulnerable Stroke Population

**DOI:** 10.1002/ana.78281

**Published:** 2026-07-24

**Authors:** Fabricio O. Lima, Francisco José de A. Mont'Alverne, Letícia C. Rebello, Daniel G. Abud, Gisele S. Silva, Michel E. Frudit, Mário B. Wagner, Jamary Oliveira‐Filho, Diogo C. Haussen, David S. Liebeskind, Octávio M. Pontes‐Neto, Norberto A.F. Frota, Guilherme Dabus, Gabriel R. de Freitas, Adson F. de Lucena, Jeffrey L. Saver, Raul G. Nogueira, Sheila C.O. Martins, for the RESILIENT Trial Investigators

**Affiliations:** ^1^ Neurology Service Hospital Geral de Fortaleza Fortaleza Brazil; ^2^ Neurointerventional Service Hospital Geral de Fortaleza Fortaleza Brazil; ^3^ Neurology Service Hospital de Base Brasília Brazil; ^4^ Department of Internal Medicine, Radiology Division Hospital das Clínicas ‐ Ribeirão Preto Medical School, University of São Paulo Ribeirão Preto Brazil; ^5^ Neurology and Neurosurgery Department Federal University of São Paulo São Paulo Brazil; ^6^ Academic Research Organization, Hospital Israelita Albert Einstein São Paulo São Paulo Brazil; ^7^ Neurointerventional Radiology Service, Federal University of São Paulo São Paulo Brazil; ^8^ Faculty of Medicine, Federal University of Rio Grande do Sul Porto Alegre Brazil; ^9^ Postgraduate Program in Health Sciences, Federal University of Bahia School of Medicine Salvador Brazil; ^10^ Department of Neurology Marcus Stroke and Neuroscience Center, Grady Memorial Hospital, Emory University School of Medicine Atlanta GA USA; ^11^ Department of Neurology and Comprehensive Stroke Center University of California Los Angeles David Geffen School of Medicine Los Angeles CA USA; ^12^ Department of Neurosciences and Behavioral Sciences Hospital das ClínicasRibeirão Preto Medical School, University of São Paulo Ribeirão Preto Brazil; ^13^ Department of Post‐Graduate Medical Sciences University of Fortaleza Fortaleza Brazil; ^14^ Miami Cardiac and Vascular Institute and Baptist Neuroscience Center Miami FL USA; ^15^ Department of Neurology Hospital Pro‐Cardiaco Rio de Janeiro Brazil; ^16^ D'Or Institute for Research and Education, Universidade Federal Fluminense Rio de Janeiro Brazil; ^17^ Neurology Department University of Pittsburgh School of Medicine Pittsburgh PA USA

## Abstract

**Objective:**

Randomized trials from high‐income countries have shown that endovascular treatment (EVT) yields similar benefits in elderly and non‐elderly patients with large vessel occlusion stroke (LVOS). However, evidence from low/middle‐income countries remains scarce. We aimed to assess the impact of age on outcomes and determine whether age modifies the effect of EVT under the resource‐limited conditions of a public healthcare system in a developing country.

**Methods:**

Pre‐specified subgroup analysis of the RESILIENT trial, a multicenter, randomized, controlled trial in Brazil comparing EVT versus medical treatment alone in anterior circulation LVOS. Patients were categorized as non‐elderly (<70 years), elderly (≥70 years), and very elderly (≥80 years). The primary endpoint was functional independence (modified Rankin scale 0–2) at 90 days. Logistic regression was performed to identify independent predictors of outcome and to assess age as an effect modifier.

**Results:**

Among 221 randomized patients (47.1% females; median [IQR] National Institutes of Health Stroke Scale, 18 [14–21]), 89 (40.2%) were elderly and 37 (16.7%) very elderly. Increasing age was independently associated with lower odds of functional independence in the entire study population (odds ratio [OR]: 0.97; 95% confidence interval [CI]: 0.95–0.99, *p* = 0.01). Age significantly modified the treatment effect of EVT. Non‐elderly patients derived substantial benefit from EVT (OR: 4.08; 95% CI: 1.71–9.76), whereas the benefit was attenuated in elderly (OR: 0.90; 95% CI: 0.17–5.42; *p*_interaction_ = 0.03) and very elderly (OR: 0.36; 95% CI: 0.11–3.21; *p*_interaction_ = 0.03).

**Interpretation:**

The effect of EVT appears significantly diminished among elderly patients in a public healthcare system in a developing country. Further studies are needed to understand the mechanism underlying these findings and properly assess the efficacy of EVT in this vulnerable population. ANN NEUROL 2026;100:644–652

Age is among the most important predictors of outcome in acute ischemic stroke (AIS).^1^ The global elderly population is expanding rapidly, with an estimated 703 million persons age 65 years or over in 2019—a number expected to double to 1.5 billion by 2050.^2^ As the incidence of stroke approximately doubles with each successive decade after age 55 years,^3^ a proportional increase in AIS cases is anticipated in the upcoming years.^4^

Despite accounting for nearly 30% of all AIS, elderly patients remain under‐represented in randomized controlled trials evaluating intravenous thrombolysis (IVT) and endovascular treatment (EVT).^5^, ^6^, ^7^, ^8^, ^9^, ^10^, ^11^ Poorer outcome in the elderly may be partially attributed to a higher burden of comorbidities—such as atrial fibrillation, congestive heart failure, and cerebral small vessel disease—as well as reduced neuroplasticity.^1^ Considerable gaps in knowledge exist for stroke treatment in elderly and very elderly patients.

Although age is an independent predictor of outcome, previous trials have shown that elderly patients derive similar relative benefit from IVT compared to younger patients.^12^ After several randomized trials have shown a remarkable effect of EVT for large vessel occlusion strokes (LVO), evaluating the efficacy of EVT in elderly patients has become of specific interest, with most studies showing a similar or even nominally larger treatment relative effect size in elderly versus non‐elderly patients.^7^, ^8^, ^10^, ^11^, ^13^, ^14^, ^15^, ^16^ However, these data were exclusively derived from high‐income countries. The incidence of major cardiovascular events and case fatality rates are the highest in low‐income countries and the lowest in high‐income countries, therefore, it is reasonable to consider that the treatment effect of EVT observed in the elderly varies across the different socio‐economic realities found across the globe.^17^

In the present study, we sought to compare the treatment effect of EVT in the elderly versus non‐elderly LVO AIS populations derived from the RESILIENT trial (ClinicalTrials.gov number, NCT02216643), a representative example of the public health care system of a developing country.

## Methods

The RESILIENT trial has been described in detail elsewhere.^14^, ^16^ Briefly, RESILIENT was a multicenter, prospective, randomized, open‐label, controlled trial with blinded outcome assessment conducted in Brazil designed to assess the safety and efficacy of mechanical thrombectomy versus medical treatment alone in AIS with LVO under the less‐than‐ideal conditions associated with the public healthcare system of a developing country. Patients were assigned in a 1:1 ratio to be treated with intra‐arterial thrombectomy and guideline‐based care (thrombectomy group) or with guideline‐based care alone (control group), including intravenous alteplase for eligible patients within 4.5 hours after symptom onset. Eligible patients were older than 18 years of age, had an occlusion in the intracranial ICA and/or MCA‐M1, could be treated within 8 hours of symptom onset, had a pre‐stroke modified Rankin scale (mRS) score of 1 or less, and a baseline National Institutes of Health Stroke Scale (NIHSS) score of at least 8 points. The main exclusion criteria on imaging were evidence of a large ischemic core, as indicated by an Alberta Stroke Program Early computed tomography (CT) score of less than 6 on CT or a score of less than 5 on diffusion‐weighted magnetic resonance (MR) imaging and the presence of a “malignant profile” of collateral circulation (absence of collaterals in the CT angiography (CTA) using maximum intensity projections [MIPs]). The trial protocol was approved by the National Regulatory Agency in Research and the institutional review board at each participating site. Enrolled patients or their surrogates provided written informed consent. Intracranial CT MIPs were used to evaluate collateral circulation using a 4‐point grading system, from 0 (absent collaterals in >50% of an MCA M2 branch (superior or inferior division) territory) to 4 (increased collaterals).^18^

### Statistical Analysis

In the current analysis, patients were classified according to 2 age thresholds as non‐elderly, elderly and very elderly (<70 vs ≥70, and <80 vs ≥80 years, respectively). The primary endpoint was functional independence (mRS, 0–2) at 90 days. Univariable analysis was used to compare elderly and non‐elderly patients according to different baseline characteristics, including comorbidities, stroke severity, imaging (ASPECTS, collateral status, and site of occlusion), time to treatment, IVT as well as treatment status, reperfusion status, and time to reperfusion (Table 1). Univariable binary logistic regression was used to test the association of each variable and the mRS at 3 months (mRS dichotomized ≤2 vs >2) for the elderly group (Table 2).

**TABLE 1:** Baseline Characteristics and Key Outcomes According to Age Group

Characteristics	All (n = 221)	Non‐elderly (n= 132)	Elderly (n = 89)	*p*
Sex (F)	104 (47.1%)	53 (40.1%)	51 (57.3%)	0.01^c^
Race (Caucasian vs all others)	118 (53.4%)	61 (46.2%)	57 (64.0%)	0.01^c^
NIHSS (median, IQR)	18 [14–21]	18 [14–21]	18 [14–21]	0.94^b^
NIHSS >16	131 (59.3%)	77 (58.3%)	54 (60.7%)	0.78^c^
ASPECTS (mean ± SD)	7.8 ± 1.3	7.6 ± 1.4	8.1 ± 0.98	0.001^a^
ASPECTS >7	153 (69.2%)	89 (67.4%)	64 (71.9%)	0.48^c^
Hypertension	145 (65.6%)	79 (59.8%)	66 (74.2%)	0.02^c^
Diabetes	60 (27.1%)	34 (25.8%)	26 (29.2%)	0.54^c^
Dyslipidemia	55 (24.9%)	29 (22.0%)	26 (29.2%)	0.15^c^
Smoking (current)	39 (17.6%)	31 (23.5%)	8 (9.0%)	0.01^c^
Alcohol abuse	37 (16.7%)	29 (22.0%)	8 (9.0%)	0.01^c^
Heart failure	43 (19.4%)	23 (17.4%)	20 (22.5%)	0.39^c^
Ischemic heart disease	30 (13.6%)	15 (11.4%)	15 (16.8%)	0.16^c^
Atrial fibrillation	27 (12.2%)	10 (7.6%)	17 (19.1%)	0.01^c^
Stroke/TIA	25 (11.3%)	17 (12.9%)	8 (9.0%)	0.39^c^
Chronic renal failure	7 (3.2%)	4 (3.0%)	3 (3.4%)	1.00^d^
Cardioembolic TOAST	93 (42.1%)	49 (37.1%)	44 (49.4%)	0.07^c^
Site of occlusion (ICA)	42 (19.0%)	23 (17.4%)	19 (21.3%)	0.49^c^
CTA collateral score (mean), n = 210	2.33 ± 0.8	2.22 ± 0.77	2.49 ± 0.70	0.02^b^
Median (IQR)	3 [2–3]	2 [2–3]	3 [2–3]	0.01^b^
1	36 (17.1%)	26 (20.8%)	10 (11.8%)	0.01^c^
2	68 (32.4%)	45 (36.0%)	23 (27.1%)	
3	106 (50.5%)	54 (43.2%)	52 (61.2%)	
IV thrombolysis	155 (70.1%)	92 (69.7%)	63 (70.8%)	0.89^c^
Final TICI, endovascular treatment arm (n = 111)				0.05^c^
TICI‐3	29 (26.1%)	16 (24.6%)	13 (28.3%)	
TICI‐2c	25 (22.5%)	11 (16.9%)	14 (30.4%)	
TICI‐2b	37 (33.3%)	26 (40.0%)	11 (23.9%)	
TICI‐2a	17 (15.3%)	10 (15.4%)	7 (15.2%)	
TICI‐1	0	0	0	
TICI‐0	3 (2.7%)	2 (3.1%)	1 (2.2%)	
TICI2b3	91 (82%)	49/62 (79%)	42/49 (85.7%)	0.36^c^
Metrics				
Time symptom‐to‐randomization,				
Mean ± SD min	239 ± 87	239 ± 86	238 ± 90	0.96^a^
Median, IQR, min	237 [171–295]			
Time symptom‐to‐randomization >240 min	105 (47.5%)	65 (49.2%)	40 (44.9%)	0.53^c^
Time symptom‐to‐vessel open, min (thrombectomy arm)				
Mean ± SD min	340 ± 93	340 ± 92	340 ± 95	0.98
Median, IQR, min	317 [272–394]			

ASPECTS = Alberta Stroke Program Early CT Score; F = female; CTA = computed tomography angiography; ICA = internal carotid artery; IQR = interquartile range; IV = intravenous; NIHSS = National Institutes of Health Stroke Scale; SD = standard deviation; TIA = transient ischemic attack; TICI = Thrombolysis in Cerebral Infarction; TOAST = Trial of Org 10172 in Acute Stroke Treatment; yr = year.

^a^ Independent samples *T* test.

^b^ Mann Whitney *U* test.

^c^ χ^2^ test.

^d^ Fisher's exact test.

**TABLE 2:** Predictors of Good Outcome in Elderly Patients

Characteristics	OR (95% CI)
Age (yr)	0.97 (0.88–1.06)
Sex (F)	0.52 (0.18–1.48)
Race (Caucasian)	0.63 (0.20–1.95)
NIHSS (mean ± SD)	0.84 (0.74–0.96)
NIHSS >16	0.32 (0.11–0.94)
ASPECTS (mean ± SD)	1.33 (0.77–2.48)
ASPECTS >7	1.96 (0.40–9.55)
Hypertension	3.04 (0.64–14.50)
Diabetes	0.43 (0.11–1.64)
Dyslipidemia	1.88 (0.61–5.79)
Smoking (current)	–
Alcohol abuse	0.72 (0.18–2.82)
Heart failure	0.71 (0.18–2.78)
Ischemic heart disease	1.63 (0.44–6.02)
Atrial fibrillation	1.32 (0.37–4.37)
Stroke/TIA	1.35 (0.25–7.34)
Cardioembolic TOAST	1.03 (0.36–2.89)
ICA occlusion	0.17 (0.02–1.40)
CTA collateral score	
Median, IQR	2.64 (0.94–7.39)
Colateral score ≥3	2.67 (0.79–8.96)
IV thrombolysis	0.78 (0.26–2.37)
Endovascular treatment	0.92 (0.32–2.58)
TICI2b3 (endovascular treatment arm, n = 111)	1.25 (0.35–4.44)
Metrics	
Time symptom‐to‐randomization, min (mean ± SD)	1.00 (0.99–1.01)
Time symptom‐to‐randomization >240 min	0.73 (0.25–2.11)
Time symptom‐to‐vessel open (thrombectomy arm) (mean ± SD)	1.00 (0.99–1.01)

ASPECTS = Alberta Stroke Program Early CT Score; CI = confidence interval; CTA = computed tomography angiography; F = female; ICA = internal carotid artery; IQR = interquartile range; IV = intravenous; NIHSS = National Institutes of Health Stroke Scale; OR = odds ratio; SD = standard deviation; TIA = transient ischemic attack; TICI = Thrombolysis in Cerebral Infarction; TOAST = Trial of Org 10172 in Acute Stroke Treatment; yr = year.

Continuous variables were reported as mean ± standard deviation (SD) or median ± interquartile range (IQR). Categorical variables were reported as absolute numbers and proportions. Differences in continuous variables were assessed by the independent‐samples T‐test or the Mann–Whitney *U* test in case of non‐normally distributed data. Differences between proportions were assessed by the χ^2^ test or the Fisher's exact test as appropriate.

Binary logistic regression modelling was performed to identify independent predictors of outcome. Critical confounding factors were chosen a priori to be included in the final model (age, admission NIHSS, IVT, site of occlusion, and treatment group) (Table 3A). Analysis of age and treatment group as an interaction term, was also performed to test whether age is an effect modifier of EVT. To minimize the risk of overfitting, least absolute shrinkage and selection operator (LASSO) regression was used to select variables for the final model. ASPECTS score was analyzed as a dichotomous variable in the logistic regression model (≤7 vs >7). Age was tested as a continuous variable in the first logistic regression model (Table 3A). To simplify clinical interpretation and applicability, age was also tested as a categorical variable. Two thresholds of age were used in the final model to examine how the effect of age would change from the elderly (<70 vs ≥70 years) to the very elderly (<80 vs ≥80 years) as both a prognostic factor and treatment effect modifier (Table 3B). As a sensitivity analysis, additional logistic regression models were also performed to examine the effect of mechanical thrombectomy using different endpoints (mRS dichotomized ≤3 vs >3; and mRS as an ordinal variable) (Tables S1 and S2, respectively). For the ordinal logistic regression analysis, the test of parallel lines was used to assess the proportional odds assumption.

**TABLE 3:** Final Multivariable Binary Logistic Regression Models for Good Outcome at 3 Months (mRS ≤2, Adjusted for Age, Baseline NIHSS, ASPECTS >7, Site of Occlusion, IV Thrombolysis, and Collaterals Score)

A—Age as a continuous and as a categorical variable without interaction terms						
Characteristics	Age (continuous‐yr)		Elderly (<70 vs ≥70 yr)		Very elderly (<80 vs ≥80 yr)	
	OR (95% CI)	*p*	OR (95% CI)	*p*	OR (95% CI)	*p*
Age (yr)	0.97 (0.95–0.99)	0.01	0.54 (0.26–1.11)	0.09	0.55 (0.20–1.47)	0.23
EVT	2.45 (1.23–4.91)	0.01	2.37 (1.20–4.68)	0.01	2.40 (1.21–4.76)	0.01
NIHSS	0.86 (0.80–0.93)	<0.001	0.86 (0.80–0.93)	<0.001	0.86 (0.78–0.93)	<0.001
Site of occlusion (ICA)	0.28 (0.10–0.82)	0.02	0.27 (0.09–0.77)	0.01	0.29 (0.10–0.84)	0.02
ASPECTS (>7)	1.28 (0.60–2.73)	0.52	1.12 (0.54–2.35)	0.75	1.05 (0.51–2.16)	0.90
Collaterals score	1.31 (0.86–2.00)	0.21	1.37 (0.90–2.08)	0.14	1.42 (0.94–2.16)	0.09
IV thrombolysis	1.13 (0.54–2.34)	0.74	1.10 (0.54–2.24)	0.80	1.15 (0.56–2.38)	0.67

B—Age as a categorical variable considering the elderly and very elderly individual thresholds with age and treatment allocation as an interaction term						
Characteristics	Elderly (≥70 yr)			Very elderly (≥80 yr)		
	OR (95% CI)	*p*		OR (95% CI)	*p*	
EVT (non‐elderly)	4.08 (1.71–9.76)	0.002	0.03*	3.29 (1.55–6.95)	0.002	0.03*
EVT (elderly/very elderly)	0.90 (0.17–5.42)	0.91		0.36 (0.11–3.21)	0.36	
NIHSS	0.86 (0.79–0.93)	<0.001		0.85 (0.79–0.92)	<0.001	
Site of occlusion (ICA)	0.28 (0.09–0.80)	0.02		0.26 (0.09–0.76)	0.01	
ASPECTS (>7)	1.22 (0.57–2.62)	0.61		1.11 (0.53–2.33)	0.78	
Collaterals score	1.38 (0.91–2.10)	0.13		1.40 (0.92–2.13)	0.11	
IV thrombolysis	1.18 (0.57–2.46)	0.65		1.19 (0.57–2.49)	0.63	

ASPECTS = Alberta Stroke Program Early CT Score; CI = confidence interval; EVT = endovascular treatment; ICA = internal carotid artery; IQR = interquartile range; IV = intravenous; mRS = modified Rankin scale; NIHSS = National Institutes of Health Stroke Scale; OR = odds ratio; SD = standard deviation; yr = year.

* *p* for the interaction term.

A *p*‐value <0.05 was considered statistically significant. IBM SPSS version 25.0 software and R software version 4.5.1 (*glmnet* package version 4.1–10) were used to perform the statistical analyses. This report followed the Strengthening the Reporting of Observational Studies in Epidemiology) STROBE guidelines.^19^

## Results

### Comparison of Baseline Characteristics and Outcomes across Age Groups

Among 221 randomized patients (47.1% females; age 66 years [IQR: 53–75] baseline NIHSS 18, [IQR: 14–21]), 89 (40.2%) were classified as elderly and 37 (16.7%) as very elderly. As compared to non‐elderly, elderly patients were more frequently females (42.7 vs 59.8%, *p* = 0.01), Caucasians (46.2 vs 64.0%, *p* = 0.01) had higher admission ASPECTS (8.1 vs 7.6, *p* = 0.001) and better collateral scores (3 vs 2, *p* = 0.01). The frequency of comorbidities was generally higher in the elderly with significant higher proportions of hypertension (75.9 vs 60.3%, *p* = 0.02) and atrial fibrillation (19.8 vs 7.8%, *p* = 0.01). Elderly were also less frequently alcohol abusers (9.2 vs 22.7%, *p* = 0.01) and current smokers (9.2 vs 24.0%, *p* = 0.01). The proportion of IVT use (70.8 vs 69.7%, *p* = 0.89) and the frequency of successful endovascular reperfusion (TICI 2b‐3, 85.7 vs 79.0%, *p* = 0.36) were similar across elderly and non‐elderly patients. Time from symptom onset to randomization and time from symptom onset to recanalization were also similar (Table 1).

Functional independence was observed in 32.6% and 20.2% of non‐elderly and elderly patients, respectively. There was no significant difference in terms of symptomatic intracranial hemorrhage (7–5.3% vs 7–7.9%; *p* = 0.57). Mortality at 90 days was higher in the elderly patients (29–22.0% vs 31–34.8%; *p* = 0.04).

### Analysis of Predictors of Functional Independence (mRS, 0–2)

In general, clinical and imaging characteristics present the same odds ratio (OR) for functional independence in the non‐elderly and elderly patients. On univariable analysis, higher age was associated with worse outcome (OR: 0.97 [95% confidence interval (CI): 0.95–0.99] per year, *p* = 0.002) and the elderly group had almost half the odds for functional independence as compared to the younger patients (OR: 0.53 [95% CI: 0.28–0.99], *p* = 0.04). In elderly patients, higher admission NIHSS (OR: 0.84 (95% CI: 0.74–0.96], *p* < 0.0001) was associated with lower odds of functional independence at 90 days (Table 2).

Age, baseline NIHSS, IVT, site of occlusion (ICA vs MCA‐M1), ASPECT score (≤7 vs > 7), collaterals score, and EVT were selected for the final regression model for functional independence at 90 days. Age remained significantly associated with functional independence (OR: 0.97 [95% CI: 0.95–0.99] per year, *p* = 0.01) on multivariable analysis, with elderly having half the odds for functional independence (OR: 0.54 [95% CI: 0.26–1.11], *p* = 0.09). A similar prognosis was present for the very elderly, although not statistically significant (OR: 0.55 [95% CI: 0.20–1.47], *p* = 0.23). Functional independence at 3 months was also independently associated with NIHSS (OR: 0.86 [95% CI: 0.80–0.93], *p* < 0.001), EVT (OR: 2.45 [95% CI: 1.23–4.91], *p* = 0.01), and ICA occlusion (OR: 0.28 [95% CI: 0.10–0.82], *p* = 0.02) (Table 3A).

### Age as a Modifier of the Treatment Effect of Thrombectomy on Functional Independence (mRS, 0–2)

When an interaction term was tested in the 2 models with age as a categorical variable (age categorized × EVT), a significant effect modification in terms of functional independence at 90 days was observed. Non‐elderly patients derived substantial benefit from EVT (OR: 4.08 [95% CI: 1.71–9.76], *p* = 0.002), whereas no significant benefit was detected in elderly patients (OR: 0.90 [95% CI: 0.17–5.42], *p* = 0.91; *p*_interaction_ = 0.03). A similar pattern (with a smaller point estimate) was observed for non‐very‐elderly (OR: 3.29 [95% CI: 1.55–6.95], *p* = 0.002) as compared to very elderly patients (OR: 0.36 [95% CI: 0.11–3.21], *p* = 0.36; *p*_interaction_ = 0.02) (Table 3B).

A strong trend for similar results was observed when different endpoints (mRS dichotomized ≤3 vs >3; and mRS as an ordinal variable) were used with non‐elderly patients deriving substantial more benefit when compared with elderly patients (*p*_interaction_ = 0.06 and 0.08, respectively) (Tables S1B and S2B). The proportional odds assumption was violated when using ordinal logistic regression for the very elderly patients and the results may not be reliable.

## Discussion

Consistent with the prior randomized trials of EVT, the present study reinforces the critical role of age as an independent predictor of outcome in patients undergoing EVT. Specifically, we observed that elderly and very elderly were much less likely to achieve functional independence compared to younger patients. However, in contrast to prior EVT trials, our findings suggest that age was a significant modifier of the treatment effect of thrombectomy on functional independence, with point estimates indicating a possible null effect of thrombectomy in the elderly and very‐elderly age groups in our population. Despite similar methodology to previous pivot trials, this discrepancy is likely attributable to the greater vulnerability of the patients treated in the public healthcare system of a developing country, where socioeconomic, educational, nutritional, and general health disparities are more pronounced compared to high‐income settings.

Population‐based stroke cohort studies have shown that individuals older than 70 years and older than 80 years represent approximately 40 to 50% and 30% of the overall ischemic stroke population, respectively. Yet, elderly patients remain under‐represented in clinical trials, particularly those evaluating acute reperfusion therapies. In the NINDS trial, only 7% of the patients were older than 80 years, while the ECASS‐3 trial completely excluded these patients^6^, ^20^ Although the safety and efficacy of EVT has been well established in multiple pivotal trials—including MR CLEAN, ESCAPE, EXTEND‐IA, and DAWN—which did not impose an upper age limit, other trials such as SWIFT‐Prime and DEFUSE 3 excluded patients over 85, and REVASCAT and THRACE excluded those over 80 years.^7^, ^9^, ^10^, ^11^, ^13^, ^15^, ^21^ Overall, the treatment effect size for EVT in these trials were similar or numerically higher in the elderly population. For instance, the adjusted common OR (acOR) for the ordinal shift analysis of the mRS at 90 days in the MR CLEAN trial was twice as high in the elderly patients (acOR [95% CI]: ≥80 years, 3.24 [1.22–8.62] vs <80 years, 1.60 [1.13–2.28]) with similar findings reported in the HERMES meta‐analysis (acOR [95% CI]: ≥80 years, 3.68 [1.95–6.92] vs <80 years, 2.44 (1.70–3.50]).^7^, ^22^ The ESCAPE trial was the only randomized study to show a benefit of EVT in terms of 90‐day mortality, with most of this benefit being derived from the elderly population (90‐day mRS 6 in EVT vs medical treatment: ≥80 years, 20% vs 44%; ≤80 years: 7% vs 10%).^9^ Therefore, although old age was a strong predictor of worse outcomes in these trials, it was not a treatment effect modifier with elderly patients displaying an at least equally robust benefit from EVT.

Contrary to these findings, our subgroup analysis of the RESILIENT trial demonstrates a significant interaction between age and treatment effect, with null estimates in both the elderly and very elderly patients. Interestingly, a similar pattern—although not statistically significant—was seen in REVASCAT, where the treatment effect was substantially lower in elderly (≥70 years) as compared to younger patients (OR: 0.9 [95% CI: 0.4–2.0] vs 2.5 [95% CI: 1.3–4.6], *p*_interaction_ = 0.19),^10^ despite the fact that REVASCAT excluded patients older than 80 years. Both RESILIENT and REVASCAT used less restrictive imaging inclusion criteria, which may have allowed enrollment of patients with larger infarcts, potentially diminishing treatment benefit, particularly among older individuals with less neuroplasticity and stroke recovery potential.

Another factor is the higher prevalence of small vessel disease and brain atrophy in the elderly, which may impair the accurate evaluation of areas of irreversible ischemia using non‐contrast CT and CTA in the acute setting.^23^ Additionally, broader literature in cardiovascular disease suggest that frailty and vulnerability may attenuate treatment effects in the elderly patients with lower socioeconomic status.^1^, ^17^ Chronological and biological age likely diverge across different populations, underscoring the need to adapt selection criteria accordingly. The concept of frailty—defined as a state of increased vulnerability to stressors and reduced homeostatic reserve—has been associated with adverse outcomes, and its prevalence is higher in individuals of lower socioeconomic status.^24^, ^25^

We chose functional independence as the key endpoint because we believe it represents the most clinically meaningful and powerful endpoint for this population. The dichotomous endpoint of functional independence (mRS, 0–2) may be more sensitive to age‐related differences because elderly patients—despite potentially deriving a modest benefit from thrombectomy—would presumably experience smaller shifts on the ordinal scale that are insufficient to cross the threshold into full independence. This pattern likely reflects greater frailty and reduced physiological reserve in older individuals, limiting their ability to regain independence even when technically classified as functionally independent at baseline. In addition, access to post‐stroke rehabilitation is limited within the Brazilian public health system, and these constraints may disproportionately affect elderly patients, for whom structured rehabilitation might be essential to achieving meaningful recovery. Finally, given the financial limitations of the public healthcare system and the socioeconomic vulnerability of the population it serves, palliative approaches rather than thrombectomy may be more appropriate for select patients—particularly elderly individuals unlikely to achieve satisfactory functional recovery despite successful reperfusion.

Therefore, it must be emphasized that our results do not imply that EVT does not any have a positive effect on some or even most of our elderly patients. However, they do call attention to our patient population particularities, including the potential need for stricter patient selection in the more advanced age groups compared to the criteria that have been traditionally used for the more affluent elderly patients found in the prior EVT trials. Previous cohort studies have suggested that elderly are more sensitive to any increase in stroke burden, supporting lower ischemic thresholds with higher age.^26^, ^27^ Accordingly, imaging markers of stroke burden such as ASPECTS and CTA collaterals presented larger differences between the non‐elderly and elderly group as predictors of good prognosis in our analysis. However, as the elderly are particularly vulnerable to higher stroke burden, EVT might have an even higher beneficial effect in those with higher NIHSS and more proximal intracranial occlusions and better collaterals.^28^

This principle of more selective treatment is exemplified by the DAWN trial, which required smaller baseline infarct volumes for inclusion of patients over 80 years (≤21ml) compared to younger patients (≤31ml or ≤51ml depending on stroke severity).^15^ This is a critical notion in health economics as the socioeconomic environments where the most vulnerable populations are found tend to be the same where resources are the scarcest, and this mandates higher cost‐effectiveness expectations.

Several limitations should be acknowledged. Subgroup analyses inherently carry the risk of spurious findings because of chance, limited statistical power with large CIs, and multiple statistical testing. We also cannot exclude the effect of unmeasured confounders. Therefore, our results should be considered exploratory. Nevertheless, subgroup analyses can provide clinically relevant insights and generate hypothesis for future studies. Last, as previously mentioned, it remains possible that elderly patients might still achieve more modest benefits in terms of ambulatory status (mRS, 0–3) or overall disability (mRS shift).

In conclusion, in contrast to data from high‐income countries, our findings suggest that the treatment effect size of EVT on LVOS outcomes seems to be significantly diminished among the more socioeconomically vulnerable elderly population treated in public healthcare system of a developing country. Whether adopting of more stringent EVT selection criteria tailored to this population could optimize outcomes to a point that would be more conducive to cost‐effectiveness expectations of low‐ and middle‐income countries should be appropriately investigated in future studies. Future studies should examine which factors—including frailty, access to rehabilitation, and broader social determinants—mediate the poorer outcomes observed in elderly patients in Brazil and other low‐ and middle‐income settings.

## Author Contributions

F.O.L., R.G.N., and S.C.O.M. contributed to the conception and design of the study; F.O.L., F.J.A.M., and R.G.N. contributed to writing the first draft of the manuscript and preparing the tables; F.O.L., F.J.A.M., L.C.R., D.G.A., G.S.S., M.E.F., M.B.W., J.O.F., D.C.H., D.S.L., O.M.P.N., N.A.F.F., G.D., G.R.F., A.F.L., J.L.S., and S.C.O.M. contributed to the acquisition and analysis of data.

## Potential Conflicts of Interest

F.O.L., L.C.R., G.S.S., M.B.W., J.O.F., D.C.H., N.A.F.F., and G.R.F. report no conflict of interest. F.J.A.M., D.G.A., M.E.F., D.S.L., O.M.P.N., G.D., J.L.S., R.G.N., and S.C.O.M. report consulting fees from Medtronic (the company involved manufactures the devices used in the study). J.L.S. reports owning stock options in RapidAI (the company developed the software for perfusion imaging analysis used in the selection of the patients).

## Supporting information


**Table S1.** Multivariable binary logistic regression models for secondary outcome at 3 months (mRS ≤3 – adjusted for age, baseline NIHSS, ASPECTS >7, site of occlusion, collateral score and IV thrombolysis). (A) Age as a continuous and as a categorical variable without interaction terms; (B) Age as a categorical variable considering the Elderly and Very Elderly individual thresholds with age and treatment allocation as an interaction term.
**Table S2.** Multivariable ordinal logistic regression models for secondary outcome at 3 months (adjusted for age, baseline NIHSS, ASPECTS >7, site of occlusion, collateral score and IV thrombolysis). (A) Age as a continuous and as a categorical variable without interaction terms; (B) Age as a categorical variable considering the Elderly and Very Elderly individual thresholds with age and treatment allocation as an interaction term.

## Data Availability

The data that support the findings of this study are available from the corresponding author on reasonable request.
