# Supplementary material for: Age Is an Effect Modifier of Thrombectomy Benefit in a Socioeconomically Vulnerable Stroke Population
Source: Ann Neurol. 2026 Jul 24;100(3):644–52. doi: 10.1002/ana.78281 (PMC13495903; doi:10.1002/ana.78281)
Supplement: Supplementary file 1 [file ANA-100-644-s001.docx]

**Table S1** Multivariable binary logistic regression models for secondary outcome at 3 months (mRS ≤ 3 – adjusted for age, baseline NIHSS, ASPECTS > 7, site of occlusion, collateral score and IV thrombolysis). A – Age as a continuous and as a categorical variable without interaction terms; B - Age as a categorical variable considering the Elderly and Very Elderly individual thresholds with age and treatment allocation as an interaction term.

**A**

| **Characteristics** | **Elderly**  (< 70 vs. ≥ 70 years) | | **Very Elderly**  (< 80 vs. ≥ 80 years) | |
| --- | --- | --- | --- | --- |
|  | **OR (95% CI)** | **P** | **OR (95% CI)** | **P** |
| Age | 0.62 (0.34-1.60) | 0.13 | 0.58 (0.26-1.33) | 0.20 |
| EVT | 2.68 (1.46-4.86) | 0.001 | 2.70 (1.48-4.92) | 0.001 |
| NIHSS | 0.90 (0.84-0.96) | 0.001 | 0.90 (0.84-0.96) | 0.001 |
| Site of Occlusion (ICA) | 0.45 (0.20-0.98) | 0.05 | 0.45 (0.21-1.00) | 0.05 |
| ASPECTS (>7) | 0.91 (0.47-1.76) | 0.79 | 0.88 (0.46-1.67) | 0.69 |
| Collaterals score | 1.14 (0.79-1.66) | 0.47 | 1.17 (0.81-1.70) | 0.40 |
| IV Thrombolysis | 1.39 (0.73-2.64) | 0.31 | 1.34 (0.70-2.55) | 0.37 |

**B**

| **Characteristics** | **Elderly (≥ 70 years)** | |  | |  | | **Very Elderly (≥ 80 years)** | | |
| --- | --- | --- | --- | --- | --- | --- | --- | --- | --- |
|  | **OR (95% CI)** | **P** | |  | | **OR (95% CI)** | | **P** |  |
| EVT (non-elderly) | 4.29 (1.95-9.46) | <0.001 | | 0.06* | | 2.87 (0.50-5.52) | | 0.002 | 0.61* |
| EVT (elderly / very elderly) | 1.35 (0.22-6.23) | 0.69 | |  |  | 1.89 (0.16-11.65) | | 0.49 |  |
| NIHSS | 0.90 (0.84-0.96) | 0.001 | |  | | 0.90 (0.84-0.96) | | 0.001 |  |
| Site of Occlusion (ICA) | 0.45 (0.20-0.99) | 0.05 | |  | | 0.45 (0.20-1.00) | | 0.05 |  |
| ASPECTS (>7) | 0.93 (0.48-1.81) | 0.83 | |  | | 0.88 (0.46-1.68) | | 0.70 |  |
| Collaterals score | 1.14 (0.78-1.66) | 0.49 | |  | | 1.17 (0.81-1.69) | | 0.41 |  |
| IV Thrombolysis | 1.41 (0.74-2.70) | 0.29 | |  | | 1.35 (0.71-2.58) | | 0.36 |  |

*P for the interaction term

**Table S2** Multivariable ordinal logistic regression models for secondary outcome at 3 months (adjusted for age, baseline NIHSS, ASPECTS > 7, site of occlusion, collateral score and IV thrombolysis). A – Age as a continuous and as a categorical variable without interaction terms; B - Age as a categorical variable considering the Elderly and Very Elderly individual thresholds with age and treatment allocation as an interaction term.

**A**

| **Characteristics** | **Elderly**  (< 70 vs. ≥ 70 years) | | **Very Elderly^$^**  (< 80 vs. ≥ 80 years) | |
| --- | --- | --- | --- | --- |
|  | **OR (95% CI)** | **P** | **OR (95% CI)** | **P** |
| Age | 0.49 (0.29-0.83) | 0.01 | 0.42 (0.21-0.83) | 0.01 |
| EVT | 2.13 (1.30-3.49) | 0.003 | 2.19 (1.33-3.60) | 0.002 |
| NIHSS | 0.90 (0.86-0.95) | <0.001 | 0.90 (0.86-0.95) | <0.001 |
| Site of Occlusion (ICA) | 0.70 (0.37-1.32) | 0.27 | 0.73 (0.39-1.38) | 0.33 |
| ASPECTS (>7) | 0.98 (0.57-1.67) | 0.93 | 0.93 (0.55-1.58) | 0.78 |
| Collaterals score | 1.15 (0.85-1.57) | 0.37 | 1.17 (0.86-1.58) | 0.32 |
| IV Thrombolysis | 1.43 (0.84-2.43) | 0.19 | 1.37 (0.80-2.35) | 0.25 |

**B**

| **Characteristics** | **Elderly (≥ 70 years)** | | | | **Very Elderly (≥ 80 years)** | | | |
| --- | --- | --- | --- | --- | --- | --- | --- | --- |
|  | **OR (95% CI)** | **P** | |  | | **OR (95% CI)** | **P** |  |
| EVT (non-elderly) | 3.02 (1.59-5.72) | 0.001 | 0.08* | | | 2.24 (1.30-3.84) | 0.004 | 0.82* |
| EVT (elderly / very elderly) | 1.26 (0.29-4.29) | 0.70 |  |  |  | 1.92 (0.22-8.50) | 0.39 |  |
| NIHSS | 0.91 (0.86-0.95) | <0.001 |  | | | 0.90 (0.86-0.95) | <0.001 |  |
| Site of Occlusion (ICA) | 0.70 (0.37-1.32) | 0.27 |  | | | 0.73 (0.39-1.38) | 0.33 |  |
| ASPECTS (>7) | 0.98 (0.37-1.32) | 0.95 |  | | | 0.93 (0.55-1.58) | 0.79 |  |
| Collaterals score | 1.16 (0.85-1.58) | 0.34 |  | | | 1.17 (0.86-1.58) | 0.32 |  |
| IV Thrombolysis | 1.43 (0.84-2.44) | 0.19 |  | | | 1.37 (0.80-2.35) | 0.24 |  |

*P for the interaction term

**^$^** Proportional odds assumption violated.
